# Impact of Multi-Scattered LiDAR Returns in Fog

**DOI:** 10.3390/s24165121

**Published:** 2024-08-07

**Authors:** David Hevisov, André Liemert, Dominik Reitzle, Alwin Kienle

**Affiliations:** Institute for Laser Technologies in Medicine and Metrology at the University of Ulm (ILM), D-89081 Ulm, Germany; andre.liemert@ilm-ulm.de (A.L.); dominik.reitzle@ilm-ulm.de (D.R.); alwin.kienle@ilm-ulm.de (A.K.)

**Keywords:** LiDAR, autonomous driving, adverse weather, augmentation, Monte Carlo simulation, analytical solution, radiative transfer equation, time-resolved radiance

## Abstract

In the context of autonomous driving, the augmentation of existing data through simulations provides an elegant solution to the challenge of capturing the full range of adverse weather conditions in training datasets. However, existing physics-based augmentation models typically rely on single scattering approximations to predict light propagation under unfavorable conditions, such as fog. This can prevent the reproduction of important signal characteristics encountered in a real-world environment. Consequently, in this work, Monte Carlo simulations are employed to assess the relevance of multiple-scattered light to the detected LiDAR signal in different types of fog, with scattering phase functions calculated from Mie theory considering real particle size distributions. Bidirectional path tracing is used within the self-developed GPU-accelerated Monte Carlo software to compensate for the unfavorable photon statistics associated with the limited detection aperture of the LiDAR geometry. To validate the Monte Carlo software, an analytical solution of the radiative transfer equation for the time-resolved radiance in terms of scattering orders is derived, thereby providing an explicit representation of the double-scattered contributions. The results of the simulations demonstrate that the shape of the detected signal can be significantly impacted by multiple-scattered light, depending on LiDAR geometry and visibility. In particular, double-scattered light can dominate the overall signal at low visibilities. This indicates that considering higher scattering orders is essential for improving AI-based perception models.

## 1. Introduction

As the optical counterpart to radar, a light detection and ranging (LiDAR) system enables the precise determination of distances by emitting light pulses with time-resolved detection of the reflected pulse response. In addition to applications such as topographic mapping [1,2], the characterization of atmospheric properties [3,4] and other areas where 3D mapping of the environment is of interest [5,6], LiDAR has become increasingly important in the field of autonomous driving [7]. Complementing established sensor technologies that only yield angular resolution, such as cameras, the additional depth information provides critical value for reliable AI-based object recognition. However, a major deficiency relates to the functionality of LiDARs under adverse weather conditions such as rain, snow, or fog, where the potential for accidents is particularly elevated, necessitating special attention to ensure optimal operational safety. The presence of scattering particles in the air results in the attenuation of the light beam which, in turn, reduces the detected signal. Furthermore, the backscattered light from the surrounding environment introduces spurious peaks and additional noise [8]. This can lead to erroneous object detection and, in the worst case, may even trigger an unintended braking maneuver. The primary issue relates to the fact that most existing datasets do not adequately reflect the full spectrum of adverse weather conditions required for training the machine learning algorithms utilized for autonomous navigation. Moreover, the labeling of adverse weather data is subject to a high potential for error. To address these limitations, the augmentation of existing datasets through simulation, for instance, calculating the signal’s appearance in the presence of meteorological phenomena such as fog, offers an effective method for incorporating all weather scenarios pertinent to the real-world application into a training dataset. A variety of approaches can be found in the literature [9], including physics-based augmentation models for snow [10] and fog [8], which build upon the LiDAR model presented by Rasshofer et al. [11]. In this context, the propagation of the pulse through a scattering medium is described by a linear model, where the detected power is represented by a time-wise convolution of the emitted pulse with the pulse response of the scene [8]. However, concerning the so-called soft target contribution, this model offers only an approximate solution for the single-scattered radiance. To ensure an accurate representation of reality, it is important to investigate physical plausibility, in particular, whether and to what extent multiple-scattered light may be relevant, to assess if accounting for higher scattering orders could further improve the quality of the augmented data. Contrary to the field of autonomous driving, the influence of multiple scattering on the LiDAR signal has already been extensively investigated theoretically within the scope of atmospheric science using the Monte Carlo (MC) method [12], which provides the numerical solution to the radiative transfer equation (RTE) and is regarded the gold standard for the computational modeling of light propagation in turbid media. Further, in atmospheric applications, approximate models based on the LiDAR equation have already been proposed to account for the higher scattering orders [13], confirming its relevance. Regarding the exact analytical solutions of the time-dependent RTE in infinite space, to the best of our knowledge, the solution for the single-scattered radiance caused by an isotropic point source [14] and, from our previous work, the solution for the total radiance induced by an unidirectional light source [15] are available. Furthermore, for second-order solutions, expressions for specific configurations, e.g., the LiDAR geometry, are presented in [16]. However, no explicit representation for the infinite-space Green’s function, i.e., the fundamental solution to the double-scattered radiance in terms of a single quadrature has been given.

In this study, the influence of multiple scattering on the detected LiDAR signal in the context of autonomous driving is investigated theoretically for different fog types, visibility ranges and beam geometries using the MC method as a numerical solution to the RTE. To this end, the LiDAR configuration, together with the bidirectional path tracing technique [17], has been implemented into a self-developed GPU-accelerated MC software [18] to enable the efficient computation of the statistically unfavorable detection geometry. In addition, the analytical solution of the RTE for the time-resolved radiance is derived in terms of scattering orders in infinite space and an explicit representation for the double-scattered radiance is provided. This allows the flexibility of the MC method to be exploited while establishing a physical ground truth through validation, thereby meeting best practice standards for simulative studies. Furthermore, to ensure representative numerical results, real particle size distributions from the literature [19] were used to calculate the scattering phase functions for fog via Mie theory. The analysis of the individual scattering acts reveals that higher scattering orders can have a significant effect on the overall signal. In particular, the fog-induced peaks are mainly due to single and double-scattered light. Under low visibility conditions, the maximum caused by the double-scattered contributions can even become clearly dominant in the overall signal. Models based on single scattering would, therefore, be unable to capture both of these peaks, resulting in artificially generated training data lacking critical features of the real-world LiDAR response. Finally, since distance determination is primarily based on peak detection in the LiDAR signal, object recognition in real-world applications is likely to be degraded, ultimately leading to less robust autonomous navigation in fog.

The remainder of the paper is structured as follows. In Section 2, the derivation of the analytical solution is presented, and the particularities of the MC methodology are discussed in brief. Subsequently, in Section 3, the validation of the MC software is demonstrated using the analytical solution of the time-resolved double-scattered radiance. Subsequently, the influence of the individual scattering orders on the LiDAR signal based on the MC method is theoretically evaluated. In conclusion, in Section 4, a summary of the key findings is provided and an outlook is given on how further improvement of the extended datasets can potentially be achieved.

## 2. Materials and Methods

### 2.1. Green’s Function in Terms of Scattering Orders

This section considers the time-resolved radiance in terms of scattering orders caused by a unidirectional point source, providing an explicit representation of the double-scattered radiance. Furthermore, an infinitely extended anisotropic scattering medium is assumed with absorption and scattering coefficients denoted by $\mu_{a}$ and $\mu_{s}$, respectively. The total attenuation coefficient is defined as $\mu_{t}=\mu_{a}+\mu_{s}$ and the effect of anisotropic scattering is described by a rotationally invariant and normalized phase function of the form $f=f(\hat{s}\;\cdot \;{\hat{s}}^{\prime })$ with an anisotropy factor $g:=2\pi \int_{-1}^{1}f\left(\mu \right)\mu \;d\mu$. In this context, the direction vector $\hat{s}\in S^{2}$ is given by the representations

(1) $$\hat{s}=\left(\begin{array}{c} s_{1}\\ s_{2}\\ s_{3} \end{array}\right)=\left(\begin{array}{c} \sin\theta \cos\phi \\ \sin\theta \sin\phi \\ \cos\theta \end{array}\right)=\left(\begin{array}{c} \sqrt{1-\mu^{2}}\cos\phi \\ \sqrt{1-\mu^{2}}\sin\phi \\ \mu \end{array}\right)\;.$$

The fundamental solution (Green’s function) for the time-resolved RTE can be formally expanded in terms of the Neumann series $G=\sum_{n\geq 0}G_{n}$, with $G_{n}=G_{n}\left(x,\hat{s},{\hat{s}}_{0},t\right)$ being the solution of the recursively defined transport equation

(2) $$\frac{1}{c}\frac{\partial G_{n}}{\partial t}+\hat{s}\;\cdot \;\nabla G_{n}+\mu_{t}G_{n}=\mu_{s}\int_{S^{2}}\;f\left(\hat{s}\;\cdot \;{\hat{s}}^{\prime }\right)G_{n-1}\left(x,{\hat{s}}^{\prime },{\hat{s}}_{0},t\right)\;d{\hat{s}}^{\prime },\;n\in N,$$

where $x\in R^{3}$, *c* is the speed of light and ${\hat{s}}_{0}\in S^{2}$ indicates the direction of the unidirectional point source. The first term $G_{0}$ which corresponds with the ballistic contribution is given by

(3) $$G_{0}\left(x,\hat{s},{\hat{s}}_{0},t\right)=ce^{-\mu_{t}ct}\Theta \left(t\right)\delta \left(x-ct{\hat{s}}_{0}\right)\delta \left(\hat{s}-{\hat{s}}_{0}\right),$$

where Θ(·) denotes the Heaviside step function. Henceforth, c=1 is set due to convenience. To proceed further, both sides of (2) are multiplied by $e^{\mu_{t}t}$ and the resulting equation is integrated along the path

(4) $$\gamma :\left[0,t\right]⟶R^{4},\;\ell ⟶\gamma \left(\ell \right):=\left(\begin{array}{c} x-\left(t-\ell \right)\hat{s}\\ \ell \end{array}\right)\;,$$

under consideration of $G_{n}{|}_{t=0}=0$ for n>0. As a result, we obtain

(5) $$G_{n}\left(x,\hat{s},{\hat{s}}_{0},t\right)=\mu_{s}\int_{0}^{t}e^{-\mu_{t}\ell }\int_{S^{2}}f\left(\hat{s}\;\cdot \;{\hat{s}}^{\prime }\right)G_{n-1}\left(x-\ell \hat{s},{\hat{s}}^{\prime },{\hat{s}}_{0},t-\ell \right)\;d{\hat{s}}^{\prime }d\ell ,\;n\in N.$$

In the case of n=1, the single-scattered radiance can be expressed in the form

(6) $$G_{1}\left(x,\hat{s},{\hat{s}}_{0},t\right)=\mu_{s}e^{-\mu_{t}t}f\left(\hat{s}\;\cdot \;{\hat{s}}_{0}\right)\int_{0}^{t}\delta \left(x-\ell \hat{s}-\left(t-\ell \right){\hat{s}}_{0}\right)\;d\ell ,\;t>0.$$

Inserting this expression into the recursion (5) for n=2 leads to the double-scattered radiance

(7) $$G_{2}\left(x,\hat{s},{\hat{s}}_{0},t\right)=\mu_{s}^{2}e^{-\mu_{t}t}\int_{0}^{t}\;\;\int_{\left|y|\leq (t-\ell \right)}\;\;f\left(\hat{s}\;\cdot \;\hat{y}\right)f\left(\hat{y}\;\cdot \;\hat{s}\right)\delta (x-\ell \hat{s}-y-\left(t-\ell -|y|\right){\hat{s}}_{0})\;dyd\ell .$$

By expanding the Dirac delta function according to

(8) $$\begin{array}{cc} \delta (x-\ell \hat{s}-y-(t-\ell -|\hat{y}\left|\right){\hat{s}}_{0})= & \frac{\delta (|x-\ell \hat{s}-(t-\ell -|\hat{y}\left|\right){\hat{s}}_{0}\left|-\right|\hat{y}\left|\right)}{|x-\ell \hat{s}-(t-\ell -|\hat{y}\left|\right){\hat{s}}_{0}{|}^{2}}\\ & \times \delta \;\left(\frac{x-\ell \hat{s}-(t-\ell -|\hat{y}\left|\right){\hat{s}}_{0}}{|x-\ell \hat{s}-(t-\ell -|\hat{y}\left|\right){\hat{s}}_{0}|}-\hat{y}\right) \end{array}$$

and reconsidering the speed of light *c*, we obtain the representation

(9) $$G_{2}\left(x,\hat{s},{\hat{s}}_{0},t\right)=2c\mu_{s}^{2}e^{-\mu_{t}ct}\int_{0}^{\ell^{*}}\frac{f\left(\hat{s}\;\cdot \;{\hat{s}}_{0}+\hat{s}\;\cdot \;v\right)f\left(1+{\hat{s}}_{0}\;\cdot \;v\right)}{|x-\ell \hat{s}-\left(ct-\ell \right){\hat{s}}_{0}{|}^{2}}\;d\ell ,$$

defined for t>r/c and

(10) $$v:=2\frac{\left(ct-\ell -\left(x-\ell \hat{s}\right)\;\cdot \;{\hat{s}}_{0}\right)\left(x-\ell \hat{s}-\left(ct-\ell \right){\hat{s}}_{0}\right)}{|x-\ell \hat{s}-\left(ct-\ell \right){\hat{s}}_{0}{|}^{2}}.$$

Furthermore, the upper limit of integration is given by

(11) $$\ell^{*}:=\frac{1}{2}\frac{{\left(ct\right)}^{2}-{\left|x\right|}^{2}}{ct-x\;\cdot \;\hat{s}}.$$

### 2.2. Monte Carlo Simulation

For the most representative comparison between the analytical and numerical solution of the RTE, it is desirable to detect the radiance at exactly one point in space and a discrete detection direction within the MC simulation, as in its analytical counterpart. However, conventional MC methods often use a finite spatial and angular range for detection in this type of comparison, since the probability of an energy packet, referred to as a photon in MC terminology, contributing to the signal in the case of an infinitely small detection area and solid angle is zero. Unfortunately, such binning leads to deviations, especially in the presence of abrupt changes in radiance, since integration is formally performed in the spatial angular domain. To compensate for this effect, the detector sizes must be exceptionally small in both spatial and angular dimensions, resulting in poor statistics and consequently, enormous computing times. In order to circumvent this limitation, bidirectional path tracing [17], a technique frequently employed in computer graphics, was implemented into a self-developed GPU-accelerated MC software [18]. In our adapted scheme, for the calculation of the *n*-fold scattered radiance, a photon is always started simultaneously from the detector and the light source, whereby the detector photon is propagated only up to the first and the light source photon up to the (n−1)th scattering event. At this point, the two sub-paths are connected by a so-called shadow ray, which can be used to calculate the contribution *w* to the total radiance via

(12) $$w=\frac{1}{{l_{s}}^{2}}w_{d}f\left(\cos\theta_{d}\right)w_{l}f\left(\cos\theta_{l}\right)e^{-\mu_{t}l_{s}}\;,$$

where $l_{s}$ is the length of the shadow ray, $w_{d}$ and $w_{l}$ are the current photon weights of the detector or light source path, and $\theta_{d}$ and $\theta_{l}$ are the angles between the shadow ray and the last path element of the detector or light source photon. This implies that in an infinitely extended medium, from n=2 onwards, each photon contributes to the simulated signal. The same holds true in the presence of an extended light source or detector aperture. Consequently, this methodology was also used to investigate the significance of multiple-scattered LiDAR returns. Besides, note that for the first scattering order n=1, only the detector path emerges so that $f(\cos\theta_{l})$ is replaced by $1/2\pi (1-\cos\theta_{0})$, where $\theta_{0}$ is the half opening angle, according to the solid angle illuminated by the light source. This is equivalent to the local estimate method [18]. Furthermore, in the case of single scattering, a photon only contributes to the detection signal if the shadow ray lies within the source aperture. Finally, it is important to address that the $1/{l_{s}}^{2}$ term in Equation (12) can potentially lead to the formation of random spikes in the simulated radiance curve due to single photons where the distance between the interaction points of the two sub-paths $l_{s}$ happens to be extremely small. In such cases, the simulations were repeated with the same parameters and the minimum of both calculations was used to filter out the outliers. The remaining photon propagation follows the standard MC scheme, where details of the algorithmic implementation can be found in [18].

## 3. Results and Discussion

### 3.1. Validation of the Monte Carlo Software

**Figure 1:**
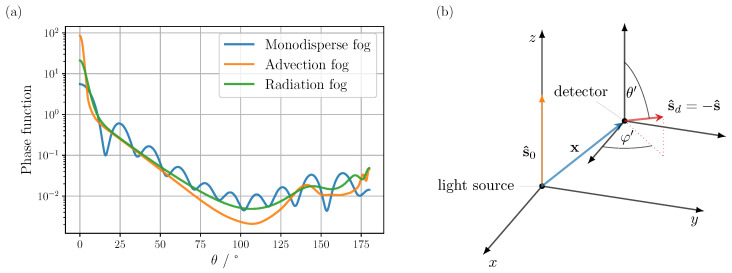
(**a**) Scattering phase functions calculated from Mie theory at a wavelength of 1550 nm for advection and a radiation fog, and a monodisperse fog, assuming a radius of r=2.5 μm for the latter case and a refractive index of 1.318 for all cases. The particle size distributions employed for the advection and radiation fog are based on values reported in [19]. (**b**) Schematic of the unidirectional source and detector geometry used for validation. The pencil beam source is located at the coordinate origin, emitting along the positive *z*-axis, with the detector located at position x. The detection direction ${\hat{s}}_{d}$ is defined by the spherical coordinates $\theta^{\prime }$ and $\varphi^{\prime }$ within the translated detector coordinate system. Since the argument $\hat{s}$ in Equation (9) is related to the direction of the incident light and not the viewing direction, ${\hat{s}}_{d}$ and $\hat{s}$ each point in exactly the opposite direction.

To confirm the physical validity of the MC code, general test cases were first compared to the analytical solution of the time-resolved double-scattered radiance presented in Section 2.1. For this purpose, the scattering phase function, see Figure 1a (blue curve), and the optical properties, $\mu_{s}=7.8\times 10^{-2}m^{-1}$ and $\mu_{a}=1.5\times 10^{-4}m^{-1}$, were calculated for a monodisperse fog with a particle radius of 2.5 μm via Mie theory, assuming a Koschmieder visibility of $V\approx \frac{3.9}{\mu_{s}}=50\;m$ and a wavelength of 1550 nm. A sketch of the unidirectional light source and detection geometry under consideration can be seen in Figure 1b.

**Figure 2:**
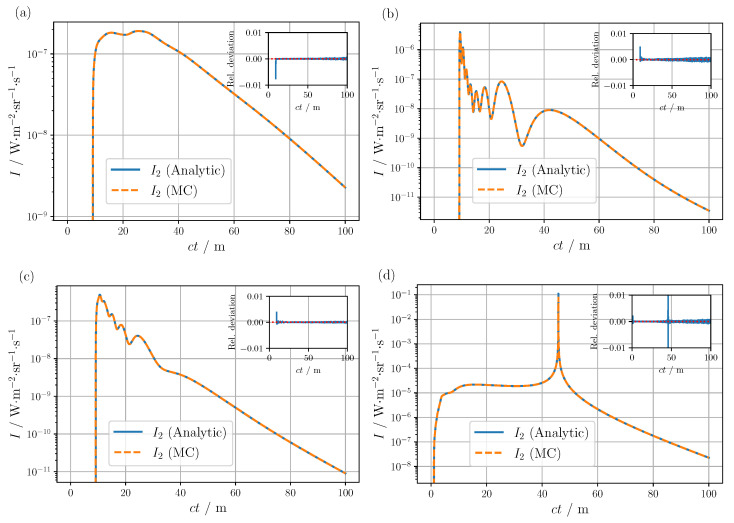
Comparison of the time-resolved double-scattered radiance obtained from the analytical solution and MC simulations for the detector directions ${\hat{s}}_{d}$ (**a**) $\theta^{\prime }=0^{\circ }$ and $\varphi^{\prime }=0^{\circ }$, (**b**) $\theta^{\prime }=180^{\circ }$ and $\varphi^{\prime }=0^{\circ }$, (**c**) $\theta^{\prime }=90^{\circ }$ and $\varphi^{\prime }=0^{\circ }$, and (**d**) $\theta^{\prime }=2.5^{\circ }$ and $\varphi^{\prime }=180^{\circ }$. The detector position is x=(3,5,7)m for (**a**–**c**), respectively, x=(1,0,0)m for (**d**). The insets show the relative deviation.

**Figure 3:**
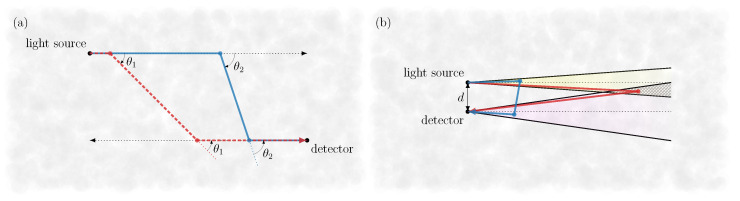
(**a**) Exemplary double scattering photon paths (red and blue) contributing to the signal in Figure 2b. Due to the special arrangement of the light source and detector, photons must be scattered twice at the same angle, e.g., $\theta_{1}$ or $\theta_{2}$, resulting in the phase function being mapped onto the radiance curve. (**b**) Sketch of the simulated bistatic LiDAR setup. In the case of low beam divergence, as in scanning LiDAR, the spatial separation between the source and detector leads to a distinct temporal separation of the detected single-scattered signal (red path) from the higher scattering orders, e.g., the double-scattered contributions (blue path).

In the first three test cases, see Figure 2a–c, where the radiance was evaluated at the detection point x=(3,5,7)m to break possible symmetries, it is evident that the MC can reproduce the result of the analytical solution even for extremely variable radiance characteristics. In particular, even the highly complex radiance response caused by the phase function in Figure 2b,c could be mapped simulatively, achieving an overall accuracy of well below 1% for a total number of simulated photons of about $10^{12}$. Notably, the oscillations in Figure 2b result from the unique arrangement of detector and light source, in which detectable photon paths are forced to scatter twice at the same angle, e.g., $\theta_{1}$ or $\theta_{2}$ as shown in Figure 3a. Since the phase function of the monodisperse fog exhibits pronounced oscillations, these also translate to the radiance at the corresponding scattering angles due to the prevailing geometric constraints on the photon trajectories. The authors note that such a geometry could provide a potential starting point for the determination of the phase function, for example, by solving the inverse problem using MC simulations. The oscillations in Figure 2c can be explained similarly. Again, a geometric restriction of the photon paths leads to a mapping from the phase function to the radiance, except in this case instead of requiring the same scattering angle twice to contribute to the signal, the sum of the two scattering angles is limited to a certain value depending on the respective path length. Finally, for the last comparison, Figure 2d, a special limiting case is considered. In contrast to the previous examples, the detector is located at x=(1,0,0)m, whereby the detection direction is inclined at a small angle towards the unidirectional light source. For this configuration, a singularity formally appears in the radiance exactly at the intersection of the detection and source directions. Therefore, due to numerical inaccuracy, a larger deviation between the simulation and the analytical solution can be observed in the area of the pole.

### 3.2. Simulations of the LiDAR Geometry

Based on the validated MC software, the influence of individual scattering orders on the overall LiDAR signal can now be analyzed on a physically sound foundation. Thus, unlike the previous tests in Section 3.1, illumination and detection are no longer assumed to be discrete, but extended over an angular range to provide a more realistic representation of a real LiDAR apparatus. Besides the contribution of the individual scattering orders, the impact of the exact LiDAR geometry is also studied in this section. Essentially, there are two primary strategies to measure the relevant field of view (FOV) [20]. One is scanning LiDAR, where, as the name suggests, a highly collimated laser beam is scanned across the FOV, providing an angular sampling of the scene. The second, flash LiDAR, involves illuminating the entire FOV and using a detector array to achieve angular resolution. Both configurations were modeled in the simulation as a bistatic LiDAR setup, as shown in Figure 3b, with a distance between the transmitter and receiver of d=2 cm. Further, according to [20], an FOV of $0.1^{\circ }$ was assumed for the detector, with a light source full aperture angle of $0.05^{\circ }$ for the scanning LiDAR and $60^{\circ }$ for the flash LiDAR. In both cases, the total emitted power was arbitrarily set to 1 W.

**Figure 4:**
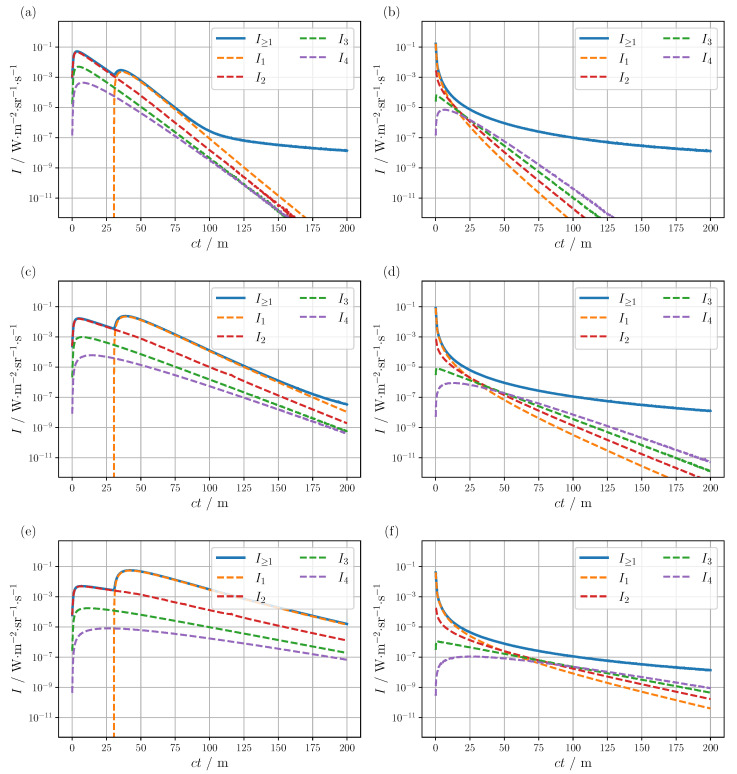
Contribution of the individual scattering orders $I_{n}$ to the total radiance for the (**a**,**c**,**e**) scanning and (**b**,**d**,**f**) flash configuration, and visibility values of 25 m, 50 m and 100 m from top to bottom. The scattering and absorption coefficient of the advection fog were calculated using Mie theory for the corresponding particle size distribution [19], yielding $\mu_{s}=7.8\times 10^{-2}m^{-1}$ and $\mu_{a}=8.4\times 10^{-4}m^{-1}$ for a visibility of 50 m. Both coefficients have been halved or doubled, for 25 m and 100 m visibility, respectively. The corresponding scattering phase function can be found in Figure 1a.

The contributions of the first four scattering orders $I_{1}$ to $I_{4}$ to the total radiance are presented in Figure 4 for both LiDAR configurations and different visibilities for an advection fog, see the orange curve in Figure 1a. Firstly, looking at the simulation results of the scanning setup, Figure 4a,c,e, depending on the visibility value, two more or less pronounced peaks can be observed in the overall radiance response. Analysis of the individual scattering orders reveals that the first peak is mainly formed by the double-scattered radiance $I_{2}$ and the second peak by the single-scattered radiance $I_{1}$. Taking a closer look at the geometrical arrangement of the light source and detector, see Figure 3b, the origin of both maxima is easily understood. Single-scattered light can only contribute to the LiDAR signal if it is scattered in the overlapping area between the light and detection cones. For higher scattering orders n>1, this geometric restriction no longer applies, allowing a signal to be registered for distances larger than *d*. As a consequence, for the scanning setup, the limited extension of the detection and light cones leads to a pronounced temporal separation of $I_{1}$ from the higher scattering orders. Note, that in contrast to the simulated bistatic configuration, no temporal separation of $I_{1}$ is anticipated for a coaxial setup due to the lack of spatial separation of detector and source (d=0 cm). The relative change in the two peak heights as a function of visibility, Figure 4a,c,e, can also be explained by simple geometric considerations. At lower visibilities, and thus larger scattering, the probability of entering the intersection region of the source and detector cones without a previous scattering event is reduced, resulting in a decrease in the peak caused by $I_{1}$ compared to the peak dominated by $I_{2}$. Conversely, for larger visibility values and a decrease in scattering, the effect is reversed and the maximum of $I_{1}$ prevails. The broadening of the $I_{1}$ peak with increasing visibility can be explained analogously. Furthermore, the different declines in the respective radiance components with varying visibility can also be attributed to a change in the scattering probability. The larger scattering present at low visibilities leads on average to a reduction in the free path length travelled between scattering events. Consequently, the probability of contributing at longer distances after only a few scattering events is reduced in comparison to larger viewing distances. The observed increase in the $I_{2}$ peak with decreasing visibility can also be attributed to a change in the mean free path length. At lower visibilities, the average distance between scattering events shortens, resulting in a higher probability of contributions at short distances, i.e., in the region of the peak, compared to higher visibilities. About the third and fourth scattering orders, it is evident for the simulated scanning setup, Figure 4a,c,e, that these generally do not make a significant contribution to the total radiance. At the lowest visibility of 25 m, see Figure 4a, starting around 100 m the entire radiance is dominated by even higher scattering orders (n>4). However, given the limited visibility and considering the signal-to-noise ratio of a real detector, this interval is unlikely to be relevant for real-world applications anyway. Altogether, the key features required for a realistic augmentation could be captured with $I_{1}$ and $I_{2}$ for the scanning arrangement assumed herein. As the peak caused by $I_{2}$ clearly dominates the overall signal in low visibility conditions, an augmentation based on single scattering models would not be a sufficiently accurate approximation of the LiDAR response, especially in this situation. Since distance determination relies on the peaks in the LiDAR signal, an AI-based perception model must also be able to deal with additional peaks caused by fog to enable reliable autonomous navigation. Models that only consider single-scattered light would only map one of the two peaks, which means that critical features that may occur in real-world applications would be lost, potentially degrading the prediction accuracy of object recognition.

Looking at the simulation results for the flash arrangement, Figure 4b,d,f, the overall signal shows a different profile compared to the scanning setup. The significant increase in the illuminated solid angle manifests within the detected signal, with the result that the radiance decreases monotonically and the peaks induced by $I_{1}$ and $I_{2}$ are no longer apparent. This change can again be related to the geometric arrangement. The extended aperture of the light source brings the overlapping area between the detector and the source into closer proximity to the receiver and transmitter, thereby enabling $I_{1}$ to contribute to the signal at an earlier stage. Theoretically, there is still some time delay in the incoming $I_{1}$ signal, which is no longer resolved within the time resolution employed. Furthermore, the full illumination of the FOV causes higher scattering orders apart from the initial peak to make up a substantial fraction of the overall signal. For example, at a visibility of 100 m $I_{4}$ already exceeds $I_{1}$ from about 70 m, see Figure 4f. Another interesting effect can be observed with variation of visibility, Figure 4b,d,f. Although the profile of the individual scattering orders changes significantly due to the change in mean free path lengths described above, the overall radiance curve remains almost unchanged except for the initial region. In summary, the early part of the signal can be mapped by $I_{1}$; however, as the distance increases, the higher scattering orders become increasingly dominant, rendering the single scattering approximation rather inaccurate.

**Figure 5:**
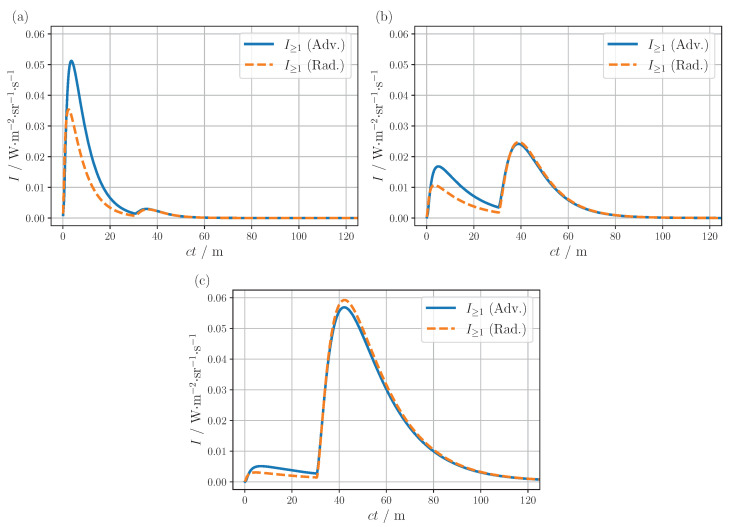
Comparison of the total LiDAR signal for advection and radiation fog at (**a**) 25 m, (**b**) 50 m and (**c**) 100 m visibility. The scattering and absorption coefficients were calculated using Mie theory for the corresponding particle size distributions [19], yielding $\mu_{a}=8.4\times 10^{-4}m^{-1}$ and $\mu_{s}=7.8\times 10^{-2}m^{-1}$ for the advection fog and $\mu_{a}=3.9\times 10^{-4}m^{-1}$ and $\mu_{s}=7.8\times 10^{-2}m^{-1}$ for the radiation fog for visibility of 50 m. Both coefficients have been halved or doubled, for 25 m and 100 m visibility, respectively. The corresponding scattering phase functions can be found in Figure 1a.

Finally, to investigate the influence of different types of fog, the total radiance of the scanning setup was compared for an advection and radiation fog (orange and green curve in Figure 1a, respectively) under different visibility conditions, see Figure 5. This time, however, a linear representation of the detector signal was chosen, as this is more in line with what a typical receiver is able to capture. The linear plots highlight once again that the total signal is governed by the two peaks mainly caused by single and double scattering, with one or the other dominating depending on the visibility as described above. Furthermore, the type of fog has a minimal impact on the form of the second peak located at about ct=40 m. This can be attributed to the geometric restriction on the photon paths and the phase functions. As also discussed above, single-scattered photons must be backscattered in the overlapping region of the detection and light cone to contribute to the signal. Since the phase functions of the two fog types exhibit comparable values within the angular range of backscattering, as illustrated in Figure 1a, only a negligible variation of $I_{1}$ is observed. In contrast, a substantially larger angular range of the phase function is relevant for double scattering, translating into a more noticeable change in the $I_{2}$ peak. Therefore, to create a realistic training data set, it may be beneficial to include different types of fog to capture the variation of the $I_{2}$ peak. The influence of the two fog types was also investigated for the flash arrangement, but apart from a change in the initial decrease, no major effect was found.

## 4. Conclusions

In this work, the influence of multiple scattered LiDAR returns in the context of autonomous driving was investigated using MC simulations to evaluate whether existing augmentation models, which only employ single scattering approximations, provide a physically plausible estimate of the light contributions from fog. To achieve efficient computation, the LiDAR geometry and the bidirectional path tracing method [17] were integrated into a self-developed GPU-accelerated MC software [18]. In the first step, the analytical solution of the RTE for the time-resolved radiance in terms of scattering orders caused by a unidirectional point source was derived and evaluated up to double scattering for validation and successfully compared with the corresponding MC implementation. For the bistatic scanning setup, it was shown that the low beam and detection aperture combined with a local separation of receiver and transmitter causes a temporal separation of $I_{1}$ from the higher scattering orders. Depending on the visibility, this results in two more or less pronounced peaks in the overall signal, which are mainly composed of single or double-scattered light. For low visibility, the double scattering can even clearly dominate the overall signal. In the case of the scanning setup, the main signal features relevant to the real-world application could be mapped via $I_{1}$ and $I_{2}$. Therefore, it may be beneficial to also consider double scattering as part of augmentation, as changes caused by $I_{2}$ can have a profound effect on the LiDAR signal and consequently on the performance of object identification algorithms. In contrast, the flash arrangement exhibits a monotonic decline in signal intensity due to the extensive illuminated FOV, with the initial portion of the curve dominated by single scattering. However, with increasing distance, higher scattering orders (n>>1) progressively dominate the overall signal, rendering the single scattering approximation increasingly inaccurate, particularly at low visibilities. It has been demonstrated that the LiDAR signal is significantly influenced by the precise beam and detection geometry, which must be considered within the augmentation model following the LiDAR technique employed. In addition to geometry, it has been shown that the fog phase function, particularly for the double-scattered radiance of the scanning arrangement, can also result in noticeable changes in the signal. Overall, the results indicate that augmentation models that only consider single-scattered light are generally not a sufficient approximation for the light contributions from fog. Since range detection and thus AI-based object identification is based on peak detection, any additional peaks caused by fog should ideally also be mapped in the LiDAR signal as part of the augmentation, so that the perception model can distinguish them from the actual object peak in a real case. Given that in the presence of fog, especially under poor visibility conditions, a peak caused by double-scattered light appears in addition to the peak consisting mainly of single-scattered light, augmentation models should also be able to map the additional peak, e.g., by including double-scattered contributions, to enable reliable navigation in fog. As the MC method allows the physically accurate simulation of arbitrarily complex LiDAR configurations and fog types, it provides an attractive option for future augmentation approaches aimed at the further improvement of AI-based perception models. To this end, extending the recently introduced integral local estimate method [18] to the spatial domain may prove to be a key factor in facilitating an even more efficient MC-based calculation of the radiance. Augmentation through the analytical solution presented might also be conceivable in settings where $I_{1}$ and $I_{2}$ provide a sufficient approximation of the total signal. In this case, however, an efficient implementation of numerical integration over the beam and detection angles would still be required. Beyond augmentation, the theoretical calculation of light propagation facilitates a more in-depth understanding of the composition of LiDAR returns under adverse weather conditions which, in turn, provides a foundation for the enhancement of LiDAR devices in the context of autonomous driving. In particular, further theoretical investigations, including polarisation, could yield valuable insights into this field of application.

## Data Availability

The raw data supporting the conclusions of this article will be made available by the authors on request.
